# D1 receptors in the anterior cingulate cortex modulate basal mechanical sensitivity threshold and glutamatergic synaptic transmission

**DOI:** 10.1186/s13041-020-00661-x

**Published:** 2020-09-05

**Authors:** Soroush Darvish-Ghane, Clémentine Quintana, Jean-Martin Beaulieu, Loren J. Martin

**Affiliations:** 1 Department of Cell and Systems Biology, University of Toronto, Toronto, ON M5S 3G5 Canada; 2 Department of Pharmacology and Toxicology, University of Toronto, 1 King’s College Circle, Toronto, ON M5S 1A8 Canada; 3 Department of Psychology, University of Toronto Mississauga, 3359 Mississauga Rd, Mississauga, ON L5L1C6 Canada

**Keywords:** Dopamine, Anterior cingulate cortex, AMPA receptors, D1 receptors, D2 receptors, CFA, Inflammation, Hypersensitivity

## Abstract

The release of dopamine (DA) into target brain areas is considered an essential event for the modulation of many physiological effects. While the anterior cingulate cortex (ACC) has been implicated in pain related behavioral processes, DA modulation of synaptic transmission within the ACC and pain related phenotypes remains unclear. Here we characterized a Crispr/Cas9 mediated somatic knockout of the D1 receptor (D1R) in all neuronal subtypes of the ACC and find reduced mechanical thresholds, without affecting locomotion and anxiety. Further, the D1R high-efficacy agonist SKF 81297 and low efficacy agonist (±)-SKF-38393 inhibit α-amino-3-hydroxy-5-methyl-4-isoxazolepropionic receptor (AMPAR) currents in the ACC. Paradoxically, the D1R antagonists SCH-23390 and SCH 33961 when co-applied with D1R agonists produced a robust short-term synergistic depression of AMPAR currents in the ACC, demonstrating an overall inhibitory role for D1R ligands. Overall, our data indicate that absence of D1Rs in the ACC enhanced peripheral sensitivity to mechanical stimuli and D1R activation decreased glutamatergic synaptic transmission in ACC neurons.

## Introduction

Dopamine (DA) binds to two classes of G protein-coupled receptor subtypes (GPCRs), classified as D1-like (D1/D5) and D2-like (D2–D4) receptors [1, 2]. A number of clinical and genetic association studies suggest that D2-like receptor mediated mechanisms have antinociceptive properties [3–6]. Yet, the role of D1-like receptors in pain modulation remains less clear. Emerging evidence has shown that ablation of spinally projecting dopaminergic neurons reduced the maintenance of long-lasting priming hyperalgesia in both sexes [7], however, spinal D5 receptors seem to play a critical role in males whereas females are more dependent on D1 receptors [8]. These studies are consistent with existing evidence supporting the pro-nociceptive effect of dopamine via a spinal D1/D5 mechanism [9]. In humans, greater functional connectivity of corticostriatal projections predict pain persistence suggesting a potential role of dopaminergic pathways in chronic pain [10]. Direct evidence from rodent studies show that dopaminergic projections from the ventral tegmental area (VTA) to the medial prefrontal cortex (mPFC) triggers antinociceptive signals [11] and optogenetic phasic activation of VTA-mPFC projections reduces mechanical hypersensitivity during neuropathic states [12].

The anterior cingulate cortex (ACC) is one of the primary cortical regions involved in the affective component of pain [13]. Human imaging studies have shown excitation of ACC neurons with noxious stimuli [14] and animals models suggest that the activity of ACC glutamatergic synapses is integral for encoding chronic pain [15, 16] and anxiety [17]. In the ACC, α-amino-3-hydroxy-5-methyl-4-isoxazolepropionic receptors (AMPARs) mediated excitatory postsynaptic currents (EPSCs) are enhanced in brain slices from animal models of chronic pain [17–21]. The ACC expresses both D1- and D2-like receptors [22, 23], with transcripts of both receptors showing a decrease following nerve injury [24]. Further, a single microinjection of DA or amantadine, a NMDA receptor antagonist into the ACC reduced autotomy behavior following nerve injury, while D1 or D2 antagonists injected into the ACC blocked the antinociceptive effects of amantadine [25]. It has also been shown that the ACC potentiates spinal cord transmission by forming direct facilitating glutamatergic synapses with spinal dorsal horn neurons (SDH), while optogenetic activation of ACC-SDH projecting neurons increased mechanical sensitivity [26].

In cortical regions such as the mPFC, D1R signaling has been shown to increase AMPAR expression in neuronal cultures [27, 28] and promote long-term potentiation of glutamatergic synapses [28]. Alternatively, D2R signaling downregulates AMPAR levels in mPFC neuronal cultures [27] and dephosphorylates AMPAR subunits necessary for induction of synaptic potentiation [28]. Whole-cell patch clamp recordings from mPFC principle neurons have demonstrated that D1Rs enhance AMPAR EPSCs [29], while D2Rs inhibit AMPAR EPSCs in the ACC [30]. Based on pharmacological studies using brain slices from the ACC, DA has been shown to inhibit AMPAR EPSCs through a D2R signaling mechanism; an effect that is enhanced by co-application of a D1R antagonist [30]. Hence, we wanted to directly determine the contribution of D1-like receptors in modulation of synaptic activity within the ACC. Furthermore, since the primary dopaminergic system connects the VTA with the limbic system and D1Rs may modulate affective sensory inputs [31], we decided to determine the contribution of D1Rs in the ACC to a pain related behavioral phenotype.

To this end, we used CRISPR/Cas9 mediated somatic gene knockout (sKO) to investigate the functional role of D1Rs in the adult ACC. Behavioral testing revealed decreased mechanical sensitivity thresholds in mice with sKO for D1Rs, while locomotion and anxiety phenotypes remained unchanged. By using whole-cell patch clamping on brain slices from mice, we observed an inhibitory role for D1-like agonists on AMPAR mediated currents, mimicking the effects of DA on EPSCs [30]. The inhibitory effect of D1 agonists was not blocked by D1 antagonists, but rather co-application of both ligands resulted in a synergistic and robust inhibition of AMPAR currents, suggesting potential novel effects for these drugs.

## Materials and methods

### Animals

Rosa26-LSL-Flag-Cas9 knockin mice (or floxCas9-EGFP mice) (Stock No: 024857, The Jackson Laboratory) [32] were used for viral injections, immunohistochemistry and behavior. Mice were maintained on a 12-h light/dark cycle with ad libitum access to food and water. Male adult (6 to 8 weeks of age) C57BL/6 J mice were acquired from the Jackson Laboratory (Bar Harbor, ME) and used for electrophysiological characterization of D1R drugs. All mice were housed in groups of 4 upon arrival and procedures were conducted in accordance with the animal care standards set forth by the Canadian Council on Animal Care (CCAC) and were approved by the University of Toronto Animal Care Committee. All animals were maintained within a temperature-controlled environment (20 ± 1 °C) with a 12: 12 h light: dark cycle. A compressed cotton nesting square and crinkled paper bedding were provided in each cage as a source of environmental enrichment. All mice had access to food (Harlan Teklad 8604) and water ad libitum.

### DNA constructs

Strategies used for CRISPR editing gene knockout (KO) have been described previously [33]. To knockout the Drd1 gene, 20-nt target sequence (*Drd1* sgRNA) in the unique exon of the gene was selected using an online CRISPR design tool (http://crispr.mit.edu/) to minimize off-target activity. For in vitro validation of the *Drd1* KO, the guide oligonucleotide was cloned into pX459 vector (Addgene plasmid # 62988). To generate sgRNA expressing AAV viral vector (pAAV *Drd1*-sgRNA-mCherry) for in vivo use, the guide was cloned into pX552 (Addgene plasmid # 60958) vector by single step cloning using SapI restriction sites.

### Cell line culture, transfection, and TIDE analysis

Neuro-2A (N2A) cells were grown in high glucose DMEM containing 10% FBS, penicillin/streptomycin and L-glutamine (HyClone-GE Healthcare, Logan, UT). Cells were maintained at 37 °C in 5% CO2 atmosphere and transfected using Lipofectamine 2000 (Thermo Fisher Scientific, Waltham, MA) according to the manufacturer’s protocols. Confluent (50–70%) N2A cells were transfected with a px459 based construct (pX459 vectors with *Drd1*-sgRNA). To select only transfected cells, 48 h after transfection cells were incubated with 3 μM puromycin for 72 h followed by 48 h incubation without puromycin. To isolate genomic DNA for TIDE analysis, cells were lysed by tail buffer (Tris pH = 8.0 0.1 M, NaCl 0.2 M, EDTA 5 mM, SDS 0.4% and proteinase K 0.2 mg/ml), and DNA was precipitated using isopropanol followed by centrifugation (13,000 *g* 15 min). DNA was resuspended in TE Buffer (10 mM Tris pH 8.0, 0.1 mM EDTA) and used for PCR to amplify guide site with: Forward primer sequence GAGGGACTTCTCCTTTCGCAT and Reverse primer sequence CCAGCAGCACACGAATACCC. PCR products were sent to sequencing with Forward primers and frequencies of mutations were determined by online TIDE tool (https://tide.nki.nl/).

### AAV viral particle preparation

For pAAV Drd1-sgRNA/mCherry, AAV serotype 5 viral particles were produced by the University of North Carolina (UNC) Vector core facility. AAV5-hSyn-mCherry and AAV5-hSyn-Cre were purchased from UNC Vector core facility (Chapel Hill, NC, US).

### Mouse stereotaxic surgery

Mice were anesthetized with a preparation of ketamine 10 mg/ml and xylazine 1 mg/ml (0.1 ml/10 g, i.p.). The animal was placed in a stereotaxic frame and the skull surface was exposed. Two holes were drilled at injection sites and 300 nl of a mixture of AAV-hSyn-Cre and AAV-hSyn-mCherry (control) or AAV-Drd1-sgRNA-mCherry was injected into the ACC (injection coordinates: AP: + 1.0; ML: ±0.3; DV: − 1.35) or into STR (injection coordinates: AP: + 1.1; ML: ±1.2; DV: − 3.7) using an injector with microsyringe pump controller (WPI) at the speed of 4 nl per second.

### Immunohistochemistry

After behavioral tests, mice were rapidly anesthetized with Avertin injection (2.5% tribromoethanol, 0.2 ml/10 g, i.p.) and transcardially perfused with 4% (w/v) paraformaldehyde in 0.1 M phosphate buffer (pH 7.5). Brains were post-fixed overnight in the same solution and stored in 4 °C. Forty microgram thick sections were cut using a vibratome (Leica, Microsystems, ON, Canada) and stored in PBS at 4 °C. Sections were mounted using fluorescence mounting medium (Dako Omnis, Agilent). Images were obtained using the EVOS M7000 Imaging System (Thermo Fisher Scientific, Massachusetts).

### Synaptosome isolation and Western blot

Synaptosomes were isolated using Syn-PER reagent according to manufacturer’s recommendation (Thermo Fisher Scientific). Briefly, dissected and frozen brain tissue was lysed in Syn-PER solution. Samples were centrifuged for 10 min at 1200 *g*. After discarding the pellet, samples were centrifuged for another 20 min at 15000 *g* to obtain synaptosomes in the pellet. The pellet was resuspended in Syn-PER solution. Protein concentration was measured by using a DC-protein assay (Bio-Rad, Hercules, CA, US). Protein extracts were separated on precast 4–20% Tris-glycine gels (Thermo Fisher Scientific, Waltham, MA) and transferred to nitrocellulose membranes. Blots were immunostained overnight at 4 °C with primary antibodies mouse anti-GAPDH (1:5000, Santa Cruz sc-322,333) and rat anti-Drd1 (1:500, Sigma d2940) Blots were incubated with secondary antibodies for 1 h at room temperature with goat anti-mouse IR Dye 680 (1:10000, Mandel 926–68,020) and goat anti-rabbit IR Dye 800 (1:10000, Mandel 926–32,211). Immune complexes were revealed using iBright Western blot imaging systems (Thermo Fisher Scientific, Waltham, MA). Quantitative analyses of fluorescent IR dye signal were carried out using Image Studio Lite 5.2 software. For quantification, GAPDH was used as a loading control for the evaluation of total protein levels.

### Behavioral tests

#### Open field test (OFT)

The OFT was performed for 30 min in an automated Omnitech Digiscan apparatus (AccuScan Instrument, Columbus, OH). Each mouse was placed in a corner of a large Plexiglas box and the total distance and the time spent in center (25% of the total surface) were recorded separately.

#### Dark-light emergence test (DLET)

The DLET was performed for 5 min with mice placed initially at the center of the dark chamber. Tests were conducted using an automated open field activity apparatus with light/dark insert (Med-Associates, St Albans, VE) with the light compartment illuminated at 800 lx. The total time spent in the light compartments was used for analysis.

#### Elevated plus maze (EPM)

EPM was performed for 5 min with mice initially placed in the center of the maze. Mice were video tracked using Viewer software (Biobserve behavior research). The time spent in the open arm was measured.

#### Y-maze test

The test was performed in a Y-maze made of opaque plastic arms. This test is based on the innate curiosity of mice to explore novel areas and presents no negative or positive reinforcers and very little stress for the mice [34]. Briefly, mice were placed into one of the arms of the maze (arm “A”) and allowed to explore the maze with one of the arms closed (new arm) for 10 min. After an hour intertrial interval, mice were returned for 5 min to the Y-maze (with all arms open) and placed into the same start arm (test trial). Mice were video tracked using Viewer software (Biobserve behavior research). The total distance travelled during the test trial and the number of visits into each arm were measured.

### Von Frey test

Mice were placed on an elevated metal mesh floor within small Plexiglas cubicles (9 × 5 × 5 cm high) and allowed to habituate for 1 h before testing began. An automated von Frey test was used (Ugo Basile Dynamic Plantar Aesthesiometer) to measure withdrawal responses. In this assay, pressure is gradually increased by the device until the mouse withdraws its hind paw; the maximal pressure at that point is displayed. Measurements were taken in both hind paws.

### CFA model of inflammatory pain

Complete Freund’s adjuvant (CFA; Sigma Aldrich, CA) was injected subcutaneously in a volume of 20 μl into both plantar hind paws using a 100-μl Hamilton syringe with a 30-gauge needle. Both paws were injected to induce bilateral activation of ACC. The automatic von Frey test was used to measure hypersensitivity of the hind paws to mechanical stimuli.

### Tissue preparation for electrophysiology

Brain slice preparations were done as previously described [30, 35]. Mice were anesthetized with 5% isoflurane and killed by decapitation. The brains were quickly removed and placed in cold (4 °C) oxygenated (95% O_2_; 5% CO_2_) artificial cerebrospinal fluid (ACSF) consisting of 124 mM NaCl, 4.4 mM KCl, 2 mM CaCl_2_, 1 mM MgSO_4_, 25 mM NaHCO_3_, 1 mM NaH_2_PO_4_, and 10 mM glucose. Brain slices (300 μm) containing coronal sections of the ACC were prepared with a VT1200S tissue slicer (Leica, Concord, ON). Slices recovered for a minimum of 60 min in a submerged holding chamber (25 °C) before recording.

### Whole-cell patch-clamp recording

Slices were removed from the holding chamber and placed in a recording chamber where they were continuously perfused with oxygenated (95% O_2_; 5% CO_2_) aCSF at a rate of 2 ml per min. Whole-cell voltage-clamp recordings from superficial layers of pyramidal neurons of the ACC region were obtained under visual guidance using a 40X objective on a Zeiss Axioskop FS upright microscope. Recordings were made with electrodes (4–6 MΩ) fabricated using a horizontal puller (P1000; Sutter, Novato, CA) and filled with an internal solution containing, 145 mM K-gluconate, 5 mM NaCl, 1 mM MgCl_2_, 0.2 mM EGTA, 10 mM HEPES, 2 mM Mg-ATP, and 0.1 mM Na_3_-GTP (adjusted to pH 7.2 with KOH). Neurons were voltage-clamped at − 60 mV using an Axon 700B amplifier (Axon Instruments, Foster City, CA), low-pass filtered at 1 kHz, and digitized at 10 kHz with Clamplex (version 10.6; Molecular Devices). Evoked EPSCs (eEPSCs) were stimulated by a tungsten bipolar stimulation electrode placed (Microprobes, Gaithersburg, MD) on the slice surface at the deep layers of the ACC proximal to the patched neuron. AMPA- and kainate-mediated eEPSCs were isolated by adding picrotoxin (100 μM) to the aCSF to block GABA_A_ (γ-aminobutyric acid type A)-receptor–mediated inhibitory synaptic currents (Additional file 1: Supplemental Figure1). For paired pulse facilitation recordings, paired stimulation (50 ms apart) was performed every 30 s. Stable baseline recordings were obtained for 5 min followed by the perfusion of pharmacological agents. Input resistance and access resistance were monitored continuously throughout each experiment; experiments were terminated if these changed by > 15%. Only recordings with stable holding current and series resistance maintained below 25 MΩ were considered for analysis.

### Pharmacological agents

The drugs used in the experiments include SKF 81297, (±) SKF 38393, R (+)-SCH-23390 hydrochloride, SCH 39166, 6-cyano-7-nitroquinoxaline-2,3-dione (CNQX) and picrotoxin. All drugs were purchased from Sigma Aldrich, CA or Tocris.

### Data and statistical analysis

Data were collected and analyzed using pClamp 10.6 software (Molecular Devices, San Jose, Ca). Data in five-minute bins were analyzed with sample traces corresponding to the averaged traces from these five-minute bins. For experiments where a washout phase after drug application was measured, one- or two-way analysis of variance (ANOVA) was used to compare the EPSCs during course of drug action versus the baseline and the washout phase. Tukey’s HSD was used for post-hoc analysis where appropriate. We used t-test comparisons to determine whether baseline and drug effects were significantly different. *p < 0.05 was considered statistically significant.

## Results

### Development of a CRISPR/Cas9 mediated somatic *Drd1* knockout strategy

We used the CRISPR/Cas9 gene editing technology to inactivate *Drd1* expression in ACC neurons of adult mice. This approach involves the use of conditional floxed-Cas9 mice [36]. Two AAVs respectively encoding a Cre recombinase or a sgRNA targeting the gene of interest are used in combination to activate Cas9 and induce a somatic gene knockout (sKO) [33]. This approach avoids developmental compensation mechanisms and allows for brain region specific manipulation of gene expression [36].

**Fig. 1:**
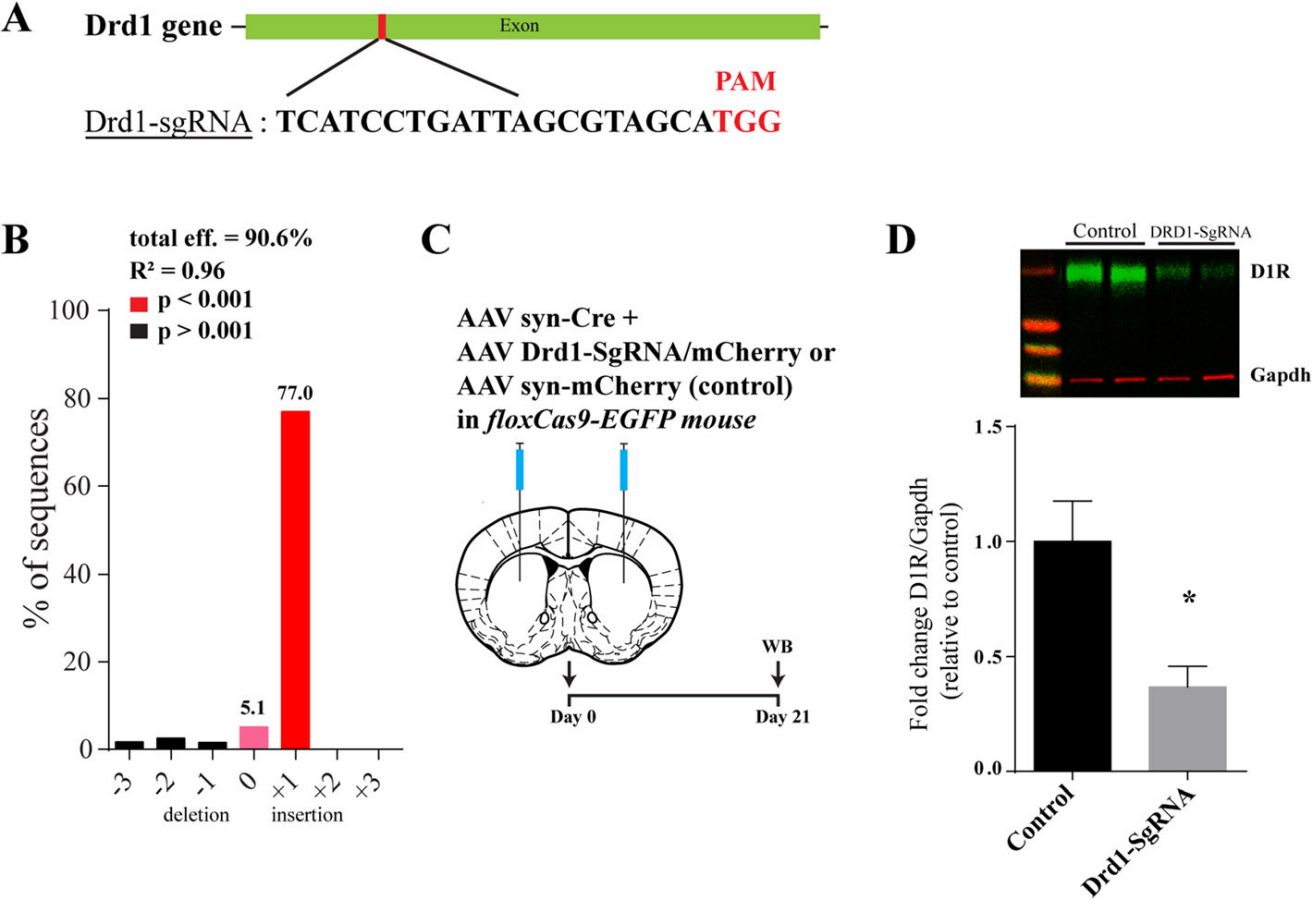
Specificity of CRISPR/Cas9 mediated somatic knockout of *Drd1* in Striatum. **a** *Drd1* targeting sequence and corresponding protospacer adjacent motif (PAM). **b** Evaluation of *Drd1* targeting sgRNA by Tracking of indels by decomposition (TIDE) analysis in Neuro2A cells. **c** Schematic diagram of experimental design. **d** Expression of *Drd1* in synaptosomes from control (*Fold change D1R/Gapdh:* 100 ± 17.57% relative to control; *n* = 5 mice) and *Drd1*-sgRNA KO striatum mice (*Fold change D1R/Gapdh*: 37 ± 9.03% relative to control; n = 5 mice) (*t*-test; t_8_ = 3.20, *p < 0.05)

First, a guide RNA (gRNA) selective for a unique exon of the D1R gene (*Drd1* sgRNA) was designed (Fig. 1a) and efficacy of the single guide RNA was measured by Tracking of indels by decomposition (TIDE) analysis in N2A cells (Fig. 1b). Multiple deletions and insertions were detected with one nucleotide insertion accounting for 77% of mutated DNA sequences (position “+ 1”). Initially, to validate the in vivo efficacy of the CRISPR/Cas9 protocol, we generated sKO of *Drd1* in the striatum, a brain region known to highly express *Drd1* [1]. A mixture of AAV Syn-Cre with AAV *Drd1*-SgRNA-mCherry (Drd1-SgRNA) or AAV Syn-mCherry (control) was microinjected into the striatum of floxCas9-EGFP mice (Fig. 1c). Three weeks following delivery of AAV constructs, D1R expression was significantly reduced in synaptosomal preparations from Drd1-SgRNA mice, relative to control mice (Fig. 1d). Hence, in vitro and in vivo delivery of CRISPR/Cas9 components efficiently knocked down the *Drd1* gene to generate brain region specific sKO for the D1R in adult mouse.

### ACC specific DRD1 somatic knockout modulates pain related behavior in adult mice

**Fig. 2:**
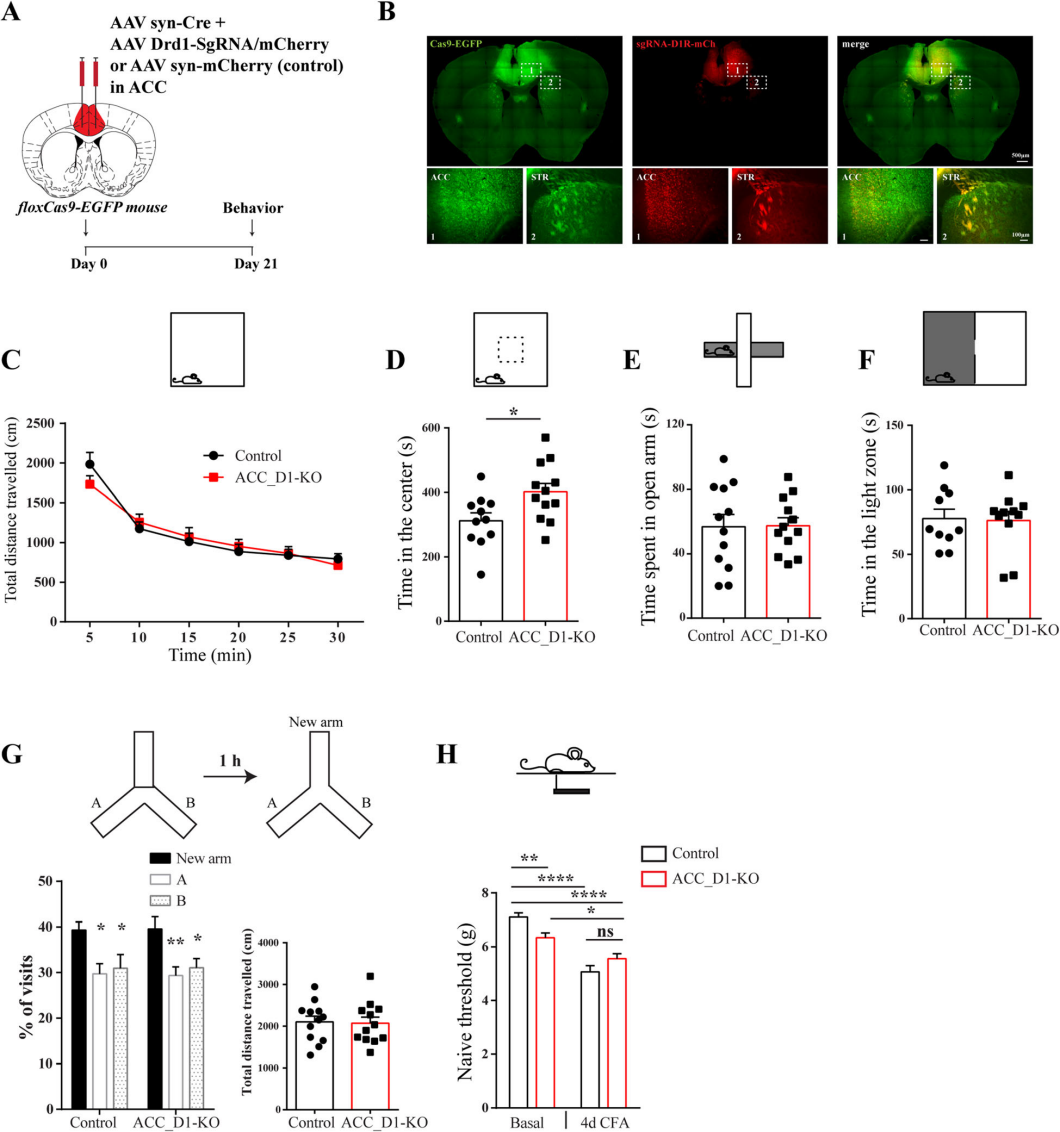
ACC CRISPR/Cas9 mediated Drd1 KO modulate pain related behaviors. **a** Schematic diagram of experimental design. **b** Representative brain slice sections obtained from Cas9-EGFP mice injected with AAV-hSyn-Cre + AAV-Drd1-sgRNA-mCherry into the ACC (dashed box 1). The striatum (dashed box 2) is shown for comparison. Scale bars = 500 μM for full brain section; 100 μM for ACC and striatum sections. **c** No difference between control and ACC_D1-KO mice in the open field for distance traveled over the 30 min observation period (two-way ANOVA, *main effect of genotype: F*_*1,22*_ = 0.01, *p* = 0.91; *main effect of time: F*_*3,67*_ *= 77.94, p < 0.0001; genotype x time interaction: F*_*1,109*_ = 1.85, *p* = 0.11). **d** ACC_D1-KO mice spend significantly more time in the center of the open field compared with control mice (t-test, *t*_*21*_ = 2.48, *p* = 0.02). **e** No difference between control and ACC_D1-KO mice in total time spent (s) in the open arms of the elevated plus maze (t-test, *t*_*22*_ = 0.07, *p* = 0.95). **f** No difference between control and ACC_D1-KO mice in the time spent in the light zone as measured using the dark/light emergence test (t-test, *t*_*19*_ = 0.15, *p* = 0.88). **g** ACC_D1-KO and control mice spend more time in the new arm of the Y-maze (left graph) compared with previously visited arms (arms A and B) (two-way ANOVA, *main effect of arm*: *F*_*2,57*_ = 10.74, *p* < 0.001; *main effect of genotype*: *F*_*1,57*_ = 0.00, *p* = 0.99; *arm x genotype interaction*: *F*_*2,57*_ = 0.01, *p* = 0.99). There was no difference between the genotypes for percentage of visits to the new arm (*t*_*19*_ = 0.07, *p* = 0.94) or total distance travelled in the Y-maze (*right graph*, *t*_*22*_ = 0.15, *p* = 0.88). **h** ACC_D1-KO mice have lower mechanical thresholds before, but not following CFA injection when compared with control mice (two-way ANOVA, *main effect of genotype: F*_*1,68*_ = 0.48, *p* = 0.49; *main effect of day*: *F*_*1,68*_ = 50.89, *p* < 0.001; *genotype x day: F*_*1,68*_ = 10.08, *p* = 0.02). All behaviors were conducted with control (*n* = 12) and ACC_D1-KO (*n* = 12) mice. Data in all graphs represent the mean and standard error of the mean (SEM). *p < 0.05, **p < 0.01, ****p < 0.0001

To investigate the functional role of D1Rs in the ACC, we delivered AAV Syn-Cre + AAV *Drd1*-SgRNA-mCherry (ACC_*D1R-KO*) or AAV Syn-mCherry (*control mice*) bilaterally into the ACC of floxCas9-EGFP mice and performed behavioral tests 3 weeks post injection (Fig. 2a). Bilateral injections were performed to avoid potential hemispheric modulation of behavior. mCherry fluorescence of AAV *Drd1*-SgRNA-mCherry was detected in cell bodies in ACC and fibers from ACC projecting neurons were present in STR confirming the specificity of the injection site into ACC (Fig. 2b). To assess whether disruption of D1R expression in the ACC changed behavior, we first measured the total distance travelled in an open field test as proxy for locomotion. No significant differences were observed between the ACC_D1R-KO and control mice over 30 min of testing (Fig. 2c). However, the time spent in the center of the open field by ACC_D1R-KO mice was significantly higher than control mice (Fig. 2d). Behavioral assessment using the elevated plus maze (EPM) as a measure of anxiety, revealed no significant difference between control and ACC_D1R-KO mice (Fig. 2e). Furthermore, in the dark-light emergence test (DLET) as an additional measure of anxiety, we found no significant difference between control and ACC_D1R-KO mice (Fig. 2f). Based on our results, ACC D1Rs do not seem to be involved in the modulation of anxiety. We also assessed working memory by using the Y-maze test [34], but found no significant difference between ACC_D1R-KO and control mice (Fig. 2g).

As a measure of pain, we assessed mechanical thresholds using automatic von Frey testing because enhanced mechanical sensitivity is consistently associated with ACC hyperactivity [17, 20, 26, 37]. In ACC_D1R-KO mice, paw withdrawal thresholds were significantly decreased compared to control mice indicating that loss of D1Rs in the ACC may increase mechanical sensitivity (Fig. 2h). Since ACC synapses are selectively potentiated in the complete Freund’s Adjuvant (CFA) model of inflammatory pain [17, 38], and D1Rs have been shown to be involved in synaptic potentiation mechanisms in the cortex [28], we tested for mechanical hypersensitivity after challenging mice with the CFA model of inflammatory pain. Four days following CFA, mechanical thresholds decreased in both ACC_D1R-KO and control mice compared to baseline responses; however, thresholds were no longer significantly different between genotypes (Fig. 2 h).

### D1-like receptor agonists inhibit eEPSCs in the ACC

**Fig. 3:**
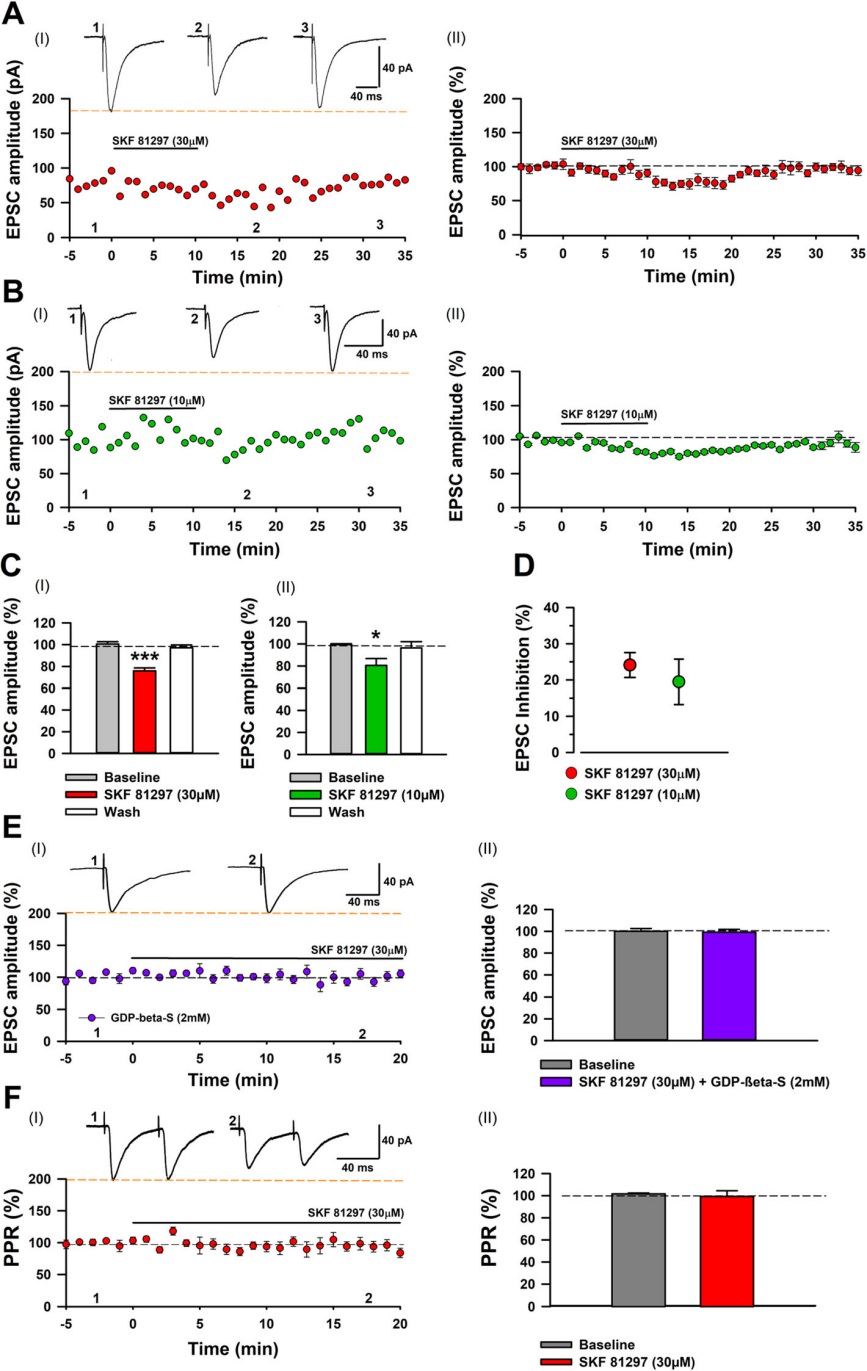
Modulation of AMPAR eEPSCs by D1R agonist SKF 81297. **a** I: Averaged sample traces of AMPAR eEPSCS and time course of SKF 81297 30 μM application. II: averaged and normalized data for time course of SKF 81297 30 μM application (*n* = 10/7 mice). **b** I: Averaged sample traces of AMPAR eEPSCS and time course of SKF 81297 10 μM application. II: averaged and normalized data for time course of SKF 81297 10 μM (*n* = 8/5mice). **c** I: EPSCs were significantly reduced by SKF 81297 30 μM compared with baseline transmission and washout phase (one-way ANOVA, *F*_*2,27*_ = 31.22, *p* < 0.001). II: EPSCs were significantly reduced by SKF 81297 10 μM compared with baseline transmission and washout phase (one-way ANOVA, *F*_*2,21*_ = 4.27, *p* = 0.02). **d** No difference in the inhibition of AMPAR mediated eEPSCs by SKF 81297 30 μM and 10 μM, (t-test, *t*_13_ = 0.62, *p* = 0.506). **e** I: Averaged sample traces and averaged and normalized data (*n* = 7/4mice). II: SKF81297 30 μM did not produce significant change in baseline transmission in presence of GDP-beta-S 2 mM in pipette (paired t-test, *t*_*12*_ = 0.43, *p* = 0.6831). **f** I: Averaged sample traces and averaged and normalized data (*n* = 8/6 mice). II: No significant difference in PPR following SKF 81297 30 μM application when compared with baseline responses (paired t-test, *t*_*7*_ = 0.52, *p* = 0.62). *p < 0.05, ***p < 0.001

Previous electrophysiology studies characterizing D1R modulation of basal AMPAR mediated transmission have yielded mixed results that seem to depend on species, brain region, neuronal subtype, concentration of drug and type of event recorded (**see** Table 1). However, since we observed that knocking down D1Rs in the ACC decreased mechanical thresholds, we postulated that D1Rs may have an inhibitory effect on ACC basal excitatory transmission. To test for this, we performed whole cell patch recording of neurons in the ACC layers II-III and V and recorded eEPSCs. After obtaining stable baseline transmission for 5 min, a high efficacy D1R agonist SKF 81297 (30 μM) [1, 53], was perfused for 10 min and subsequently washed out. The application of SKF 81297 (30 μM) significantly reduced the amplitude of eEPSCs, which returned to baseline following washout (*SKF 81297 30* μM, 75.87% ± 2.75%; *washout:* 97.17% ± 2.7%, Fig. 3a and CI). Furthermore, a lower concentration of SKF 81297 at (10 μM) inhibited eEPSC amplitudes, which also returned to baseline following washout (*SKF 81297 10* μM*:* 80.46% ± 6.27%; *washout:* 96.37% ± 5.47%, Fig. 3b and cII). The percentage of inhibition mediated by SKF 81297 30 and 10 μM is shown in Fig. 3d for comparison.

**Table 1:** Electrophysiology studies characterizing D1R modulation of basal AMPAR mediated transmission. D1R agonist modulation of basal AMPAR mediated currents

D1R agonist	Species	AMPAR current	Result	Neuron Type	Synaptic locus	Region	Reference
SKF 81297 (30 μM) DA (30 μM)	*Rat*	eEPSC eEPSC, mEPSC	Inhibit //	Magnocellular //	Presynaptic //	BF //	[39]
SKF 81297 (30 μM)	*Rat*	eEPSC	Inhibit	Cholinergic	Presynaptic	BF	[40]
SKF 81297 (1 μM)	*Rat*	eEPSC	Inhibit	Pyramidal layer V	Presynaptic	PFC	[41]
SKF 38393 (10 μM)	*Rat*	eEPSC	Enhance	Pyramidal Layer II/III	Postsynaptic	PFC	[29]
DA (250 nm-5 μM)	*Monkey*	eEPSC	Inhibit	Pyramidal layer III	Undetermined	PFC	[42]
SKF 81297 (1 μM)	*Rat*	eEPSC	Enhance	Pyramidal Layer II/lll	Postsynaptic	PFC	[43]
SKF 81297 (5 μM)	*Mice*	eEPSC	No effect	Pyramidal	–	PFC	[28]
DA (50 μM) SKF 81297 (10 μM)	*Mice* *//*	sEPSC //	Enhance //	D1R+ neurons //	Presynaptic //	Dorsal Striatum //	[44]
SKF 81297 (1 μM)	*Rat*	Kainite EPSC		Cultured MSN		Striatum	[45]
SKF 38393 (10 M)	*Rat*	AMPA EPSC	Enhance	MSN	Postsynaptic	Dorsal Striatum	[46]
SKF 38393 (5 μM) //	*Rat* *//*	eEPSC glutamate EPSC	Enhance No effect	MSN //	– –	Striatum	[47]
DA (75 μM) SKF 81297 (30 μM) SKF 38393 (100 μM)	*Rat* *//* *//*	eEPSP // //	Inhibit No effect LTD	Field & whole cell // //	Presynaptic	NAc // //	[48]
DA (50 μM) SKF 38393 (10 μM)	*Mice*	eEPSC //	Inhibit //	MSN //	Postsynaptic //	NAc //	[49]
SKF 81297 (5 μM)	*Mice*	Kainate EPSC	enhance	MSN	Postsynaptic	Neostriatum	[50]
SKF 81297 (5 μM)	*Chicken*	mEPSC	No effect	Pyramidal layer II/III Motoneurons	–	ACC	[51]
SKF 38393 (10 μM)	*Mice*	glutamate EPSC	Enhance		–	Embryonic	[52]

Studies were selected based on activity mediated by D1R pharmacological agonists and antagonist, *BF* Basal forebrain, PFC Prefrontal cortex, NAc Nucleus Accumbens, ACC Anterior Cingulat Cortex, *eEPSC* Stimulation evoked excitatory postsynaptic current, mEPSC miniature excitatory postsynaptic potential, *Kainate EPSC and AMPA EPSC* EPSC Evoked by application of kainite or AMPA, *LTD* Long-term Depression, *MSN* Medium spiny neuron

To ensure the observed inhibitory effects were not specific to SKF 81297, we used another selective D1R agonist, (±)-SKF-38393 [53]. Following a similar protocol, we briefly applied (±)-SKF-38393 (50 μM) for 10 min and subsequently washed out the drug (Additional file 1, Supplemental Figure 2A). Average and normalized data show that application of (±)-SKF-38393 (50 μM) significantly inhibited eEPSCs and washing SKF-38393 increased the eEPSC amplitudes towards the baseline ((±)-SKF-38393 *50 μM:* 78% ± 4.9%; *washout:* 96.6% ± 8.1%, Additional file 1, Supplemental Figure 2A and B). In comparing the percentage of eEPSC inhibition mediated by SKF 81297 and (±)-SKF-38393 no difference was found (*SKF 81297 30* μM, 24.1% ± 3.45%; *(±)-SKF-38393 50 μM*, 22.16% ± 5% Additional file 1, Supplemental Fig. 2C and D). In addition, a lower concentration of (±)-SKF-38393 (10 μM) exerted a moderate inhibition of eEPSCs (86.63% ± 4.4%, *n* = 5; Additional file 1, Supplemental Figure 2C, D and E). Based on these results, both D1 agonists at different concentrations have inhibitory effects on AMPAR mediated eEPSCs, similar to DA mediated inhibition of ACC AMPAR eEPSCs from a previous report [30].

### D1 agonists inhibit eEPSCs by postsynaptic GPCR action

Since the inhibitory effects of DA in the ACC are dependent on GPCR signaling in postsynaptic neurons [30], we tested whether the inhibitory effect of SKF 81297 (30 μM) involved GPCRs expressed by postsynaptic ACC neurons. eEPSCs were recorded with the addition of guanosine-5′-O-2-thiodiphosphate (GDP-beta-S) (2 mM), a broad inhibitor of GPCRs [54] in the recording pipette to block postsynaptic GPCR signaling [17]. GDP-beta-S (2 mM) in the recording pipette blocked the inhibitory effect of SKF 81297 (30 μM) on eEPSC amplitudes (SKF 81297 + *GDP-beta-S:* 99.32% ± 5.1% of baseline; Fig. 3**e**). Paired-pulse facilitation (PPF), measured as paired-pulse ratio (PPR) involves evoking two stimuli in quick succession, is considered a model of presynaptic plasticity and a measure of presynaptic function [55]. A single stimulus was delivered, followed 50 ms later by a second stimulus. Baseline eEPSCs were recorded using the paired pulse stimulation protocol and SKF 81297 (30 μM) was applied after 5 min of baseline recording. Inhibition mediated by SKF 81297 (30 μM) did not significantly change PPR relative to the baseline (97.02 ± 5.42% of baseline; Fig. 3f). Unaltered PPR and blockade of inhibition by SKF 81297 (30 μM) with GDP-beta-S (2 mM) in the pipette indicates a postsynaptic mechanism of action by SKF 81297. Also, the inhibitory effect of (±)-SKF-38393 (50 μM) had no significant effect on PPR of eEPSCs (104.42% ± 7.45% of baseline, Additional file 1, Supplemental Figure 3D**)**. Hence, both D1R ligands mediate inhibition of eEPSCs by postsynaptic mechanisms.

### D1R selective antagonists, SCH-23390 and SCH 39166 fail to block inhibition by D1 agonists

**Fig. 4:**
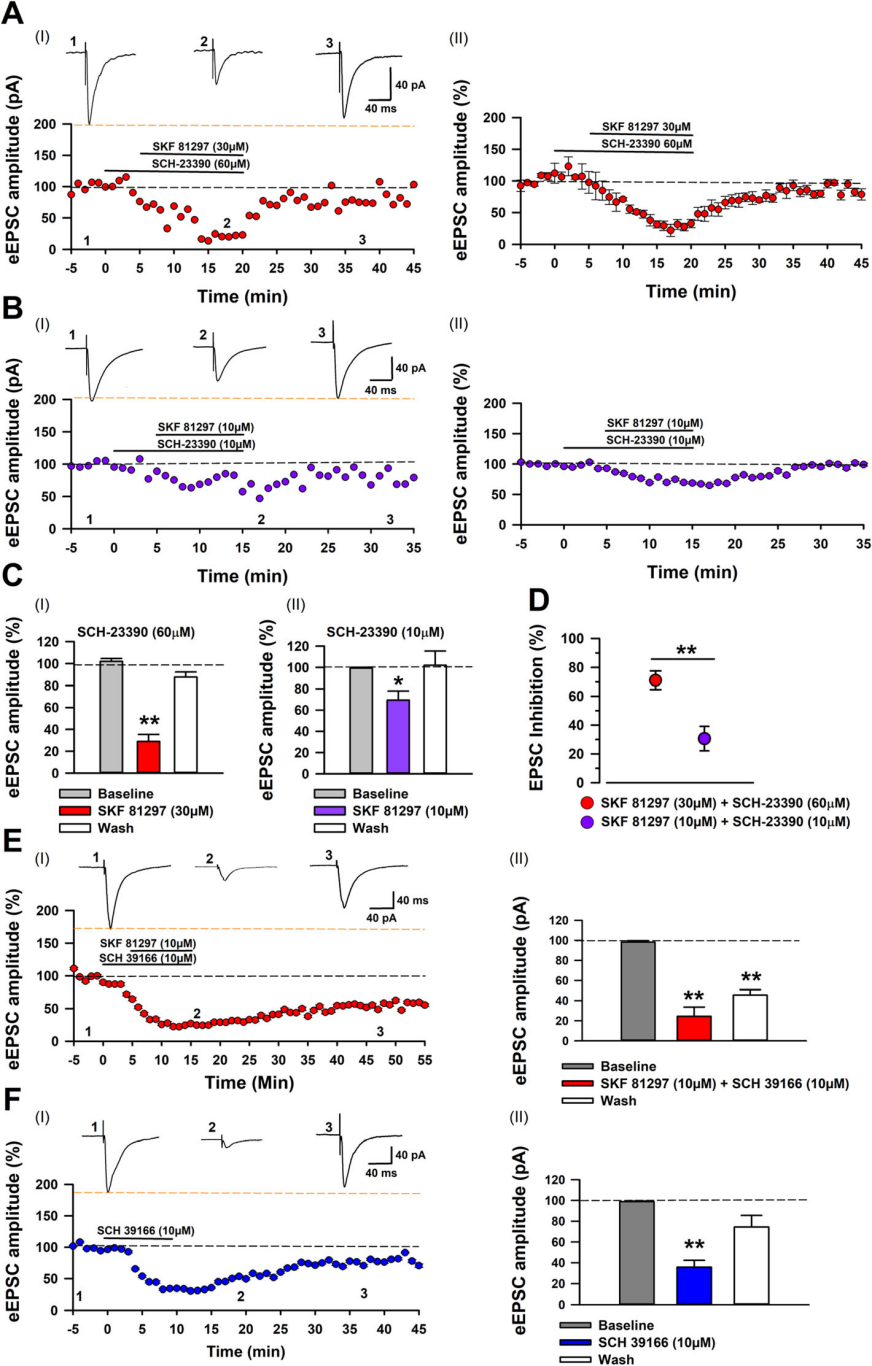
D1R antagonists do not block the inhibitory effect of SKF 81297 on eEPSCs. **a** I: Averaged traces of AMPAR mediated currents and time course for the application of the D1R antagonist SCH-23390 60 μM and the D1R agonist SKF 81297 30 μM. II: Averaged and normalized data for eEPSC amplitudes, before, during and following application of SCH-23390 60 μM and SKF 81297 30 μM (*n* = 6/5mice). **b** I: Averaged traces and time course of AMPAR mediated currents for the application of the D1R antagonist SCH-23390 10 μM and the D1R agonist SKF 81297 10 μM. II: Averaged and normalized data for eEPSC amplitudes, before, during and following application of SCH-23390 10 μM and SKF 81297 10 μM (n = 7/4 mice). **c** I: SKF 81297 30 μM produces a robust inhibition of eEPSCs when administered in the presence of the D1R antagonist, SCH-23390 60 μM, which returned towards baseline following washout (one-way ANOVA, *F*_*2,12*_ = 63.63, *p* < 0.0001). II: SKF 81297 10 μM produces a robust inhibition of eEPSCs when administered in the presence of the D1R antagonist, SCH-23390 60 μM, which returned towards baseline following washout (one-way ANOVA, *F*_*2,18*_ = 3.92, *p* = 0.03). **d** Comparison of percent inhibition of eEPSCs by SKF 81297 and SCH-23390 at different doses (independent t-test, *t*_*10*_ = 3.51, *p* = 0.006). **e** I: Averaged traces with normalized and average data (*n* = 5/3 mice). II: The D1R antagonist SCH 39166 10 μM does not block the inhibitory effect that SKF 81297 10 μM has on eEPSC amplitude. Following washout EPSCs remained depressed relative to the baseline (one-way ANOVA, *F*_*2,8*_ = 25.4, *p* < 0.0001). **f** I: Averaged traces with normalized and average data for application of SCH 39166 (*n* = 5/4 mice). II: Application of SCH 39166 10 μM alone inhibits AMPAR mediated eEPSCs, which returned to baseline following washout (one-way ANOVA, *F*_*1,5*_ = 29.89, *p* = 0.002). *p < 0.05, **p < 0.01

Since our data showed that D1-like agonists inhibit eEPSCs, we reasoned that administration of a D1-like antagonist would block these effects. For these experiments, we used the well-studied pharmacological agent SCH-23390, a known inhibitor of D1-like receptors [28, 56, 57]. We recorded baseline eEPSCs and applied SCH-23390 followed by application of SKF 81297. In previous work, a high concentration of DA antagonists was required to block DA modulation of eEPSCs in the ACC [30], therefore, we administered a higher concentration of SCH-23390 (60 μM) than SKF 81297 (30 μM). To our surprise, co-application of SCH-23390 and SKF 81297 produced a robust and significant inhibition of eEPSCs, which returned to baseline levels following washout (*SCH-23390 + SKF 81297:* 28 ± 6.46%; *washout:* 87.69 ± 6.46%; Fig. 4a and c). Similarly, SCH-23390 (60 μM) produced similar inhibition of eEPSCs when co-applied with (±)-SKF 38393 (50 μM) (35.67% ± 4.75% of baseline) and washout of drugs returned eEPSC amplitude towards baseline levels (81.77% ± 11.45% of baseline, Additional File 1, Supplemental Figure 3A and B**).**

Since the inhibition of eEPSCs by SKF 81297 was enhanced in the presence of a high dose of SCH-23390 (60 μM), we presumed that SCH-23390 may have off-target effects at high concentrations. Hence, we used SCH-23390 at a lower concentration (10 μM), which has been shown to block dopaminergic effects in PFC brain slice preparations [53, 56]. Pre-application of SCH-23390 (10 μM) failed to block inhibition of eEPSC amplitude by a low concentration of SKF 81297 (10 μM**)** (69% ± 8.49% of baseline; Fig. 4b and c). Similarly, inhibition of eEPSCs by (±)-SKF 38393 (50 μM) and a high concentration SKF 81297 (30 μM) were not blocked by SCH-23390 (*(±)-SKF 38393 50 μM +* SCH-23390 10 μM: 71.1% ± 6.66%; *SKF 81297 30 μM +* SCH-23390 10 μM*:* 85.38% ± 4.9%; Additional file 1, Supplemental Figure 3C and D).

To explore whether the inability of SCH-23390 to block the effects of SKF 81297 was specific to this D1R antagonist, we used SCH 39166, another selective D1R antagonist [58]. Co-application of SCH 39166 (10 μM) and SKF 81297 (10 μM) inhibited eEPSCs (24.4% ± 9.19% of baseline, Fig. 4e), which remained significantly depressed following washout (45.55% ± 5.38% of baseline, Fig. 4e). Given the robust inhibitory effect of this drug combination, we applied SCH 39166 (10 μM) without SKF 81297. Following a brief application of SCH 39166 (10 μM), eEPSC amplitude was significantly inhibited (35.5% ± 6.52% of baseline, Fig. 4f). However, unlike the co-application of SCH 39166 and SKF 81297, washing out SCH 39166 alone returned eEPSC amplitude back to baseline levels (*baseline:* 98.82% ± 0.74%; *washout:* 74.35% ± 11.28%, Fig. 4e). Furthermore, application of SKF 81297 (10 μM) in the presence of SCH 39166 (1 μM) significantly inhibited eEPSCs (66.82% ± 4.67% of baseline, Additional file 1, Supplemental Figure 3E). Our results thus far reveal that D1R ligands have complex pharmacology, indicative of potential mechanisms of action, independent of D1Rs in ACC brain slices.

## Discussion

Excitation of cortical brain regions is consistently observed in animal models of chronic pain [15], and enhanced AMPAR mediated eEPSCs responses are one of the established mechanisms for this observed effect [17–20, 26, 37, 59]. In the current paper, we combined emerging evidence for involvement of mesolimbic dopaminergic system in modulation of pain [60–62] with the question of how the dopaminergic system modulates AMPAR transmission in the ACC. This led us to investigate the role of an ACC dopaminergic receptor subtype in modulation of behavior and AMPAR transmission. Indirect evidence suggests that D1Rs are involved in the modulation of synaptic transmission in the ACC [30]. Hence, we wanted to specifically define a role for D1R signaling in the ACC in the context of pain. By using CRISPR/Cas9 methods, we generated a sKO for D1Rs in ACC neurons of adult mice. This method is particularly useful as it avoids developmental compensation mechanisms [36, 63]. Behavioral testing of ACC_D1R-KO mice, revealed that baseline mechanical thresholds were significantly lower in comparison to control mice, but without any alteration in locomotion, anxiety, working memory, and hypersensitivity in the CFA model of inflammatory pain. These results indicate a selective role for ACC D1Rs in modulating baseline sensory responses in pain naïve mice.

Since enhanced glutamatergic transmission in the ACC is correlated with decreased mechanical sensitivity [17, 18], decreased mechanical thresholds in ACC_D1R-KO mice suggests that D1Rs may have an inhibitory role on glutamatergic transmission in the ACC. Consistent with this idea, low and high concentrations of the D1R agonists, SKF 81297 and (±)-SKF 38393 reduced AMPAR eEPSC in ACC slices. These results are similar in nature to the mechanism of exogenously applied DA in ACC slices from a previous report [30]. However, D1Rs are Gα_s_/olf-coupled and stimulate adenylyl cyclase and cAMP production, while D2Rs are Gi-coupled and inhibit adenylyl cyclase and cAMP production [64]. This typically leads to D1Rs and D2Rs serving excitatory and inhibitory modulation of neuronal excitability, respectively [53, 65]. In the striatum and mPFC, D1Rs enhance AMPAR EPSCs through upregulation of PKA activity [29, 45, 47, 52, 66]. This upregulation is mediated by an upregulation of GluR1 AMPARs [27] and phosphorylation at Ser^845^ [28, 45], a mechanism necessary for induction of long-term potentiation in the ACC [17, 59]. D1Rs also inhibit AMPAR EPSCs in the mPFC [41] and coactivation of D1- and D2Rs inhibit EPSCs in the primate mPFC, an effect that is blocked by the D1R antagonist SCH-23390 [42]. Likewise, in the ACC, PKA activity upregulates synaptic AMPAR transmission through insertion of calcium permeable AMPARs and phosphorylation of GluR1 at Ser^845^ [17, 21, 59, 67]. Further, a traditionally Gα_s_-coupled receptor, such as the D1R is also capable of neuronal inhibition through N-type Ca^2+^ channel modulation indicating that D1Rs may display promiscuity in their Gα subunit coupling [68]. In neostriatal neurons, activation of D1Rs inhibit N-type calcium channel activity via a PKA-dependent pathway [69], while new data suggest that D1R inhibition of neurons in the ACC may occur through modulation of hyperpolarization-activated cyclic nucleotide–gated (HCN) channels [70].

An inhibitory role for D1Rs is contradictory to the pro-excitatory properties of D1R signaling as shown in previous work on PFC neurons [28, 29]. Studies on ACC synaptic plasticity in chronic pain have exclusively focused on long-term potentiation as the mechanism for encoding long-lasting pain [15, 16, 59]. Based on our results, we observe an inhibitory effect by D1R agonists on basal transmission and the reduction of basal sensory thresholds in ACC_D1R-KO mice, making it probable that D1Rs exert inhibition of basal AMPAR transmission. In support of this, a recent study has shown that ACC neurons form direct synapses with SDH to modulate mechanical sensitivity through glutamatergic synapses [26]. Hence, modulation of ACC descending facilitatory projections by D1Rs is a possible mechanism of action. Further research is required to explore the modulation of ACC-SDH projecting neurons by D1Rs.

In our experiments and previous work, D1R agonists and dopamine inhibit AMPAR EPSC transmission by an average of 20–25% [30]. Thus, we speculate that the moderate change in pain sensitivity observed in the ACC_D1R-KO mice is physiologically appropriate and expected rather than a larger effect. However, it is important to appreciate that the pain phenotype we have observed is novel and unique. This is the first time that the D1 receptor is specifically downregulated in the ACC of adult mice. In addition, this is the first time a DA receptor subtype in a cortical region has been shown to be a modulator of basal peripheral sensitivity to mechanical stimuli. Thus far, studies on ACC glutamatergic transmission and pain have focused on plasticity mechanisms in response to injury [15, 17–19, 28], leaving basal synaptic transmission without a well-defined functional role. The phenotype of ACC_D1R-KO mice warrants further research, especially toward exploring dopamine receptor signaling in the sensory and affective modulation of pain.

Our current experiments used a sgRNA targeting Drd1 as a “knockout condition” where gene expression was knocked down (e.g. 80% reduction of gene expression) in affected cells, however this approach also generates a controled mosaic of genome disruption. So, if the expression of the gene is knocked out in affected cells, the gene structure may be perfectly normal in adjacent cells. This approach is a not a knockout or knock-in in the classical sense of what would be achieved using a germinal manipulation, but represents a somatic knockout and intersectional (a more neural circuit selective method) knockout as previously described by us [36, 63, 71]. Although the ACC has a well-defined role in anxiety, ACC_D1R-KO mice do not seem to have a robust change in anxiety behavior, except for more time spent in the center of the open field. This prompted us to test these mice on two other tests of anxiety (EPM and DLET), which did not reveal any further differences. In the published literature there is a poor correlation between performance in the OFT and EPM test, while EPM and DLET exhibit a greater correlation [72]. In addition, OFT correlates well with freezing responses in contextual fear conditioning, while EPM does not suggesting that the OFT may be a more useful metric of locomotor activity [73].

Despite our results with D1R agonists and their similarity in function to DA [30], we cannot conclusively claim that inhibition by SKF 81297 and (±)-SKF 38393 are mediated exclusively by D1Rs. The inhibitory effects of SKF 81297 and (±)-SKF 38393 were not blocked by SCH 23390 and SCH 39166, two widely used D1R antagonists. Co-application of D1-like agonists (i.e. high concentration of either SKF 81297 or (±)-SKF-38393) in combination with a high concentration of SCH 23390 (a D1-like antagonist) produced a robust synergistic depression of AMPAR currents in ACC slices, similar to a synergistic depression induced by SCH 23390 and DA in a previous report [30]. Since, SCH 23390 also acts as an agonist for 5HT2c receptors [74–76], which inhibit AMPAR eEPSCs in the ACC [77], one possibility for the observed synergistic depression of AMPA currents is non-specific binding of SCH 23390 to serotonin receptor subtypes. These results further emphasize the need for pharmacological studies on genetically defined D1R+ neurons [53]. However, it is important to note that the D1R antagonist, SCH 39166 is clinically effective in treating tics related to pediatric [78] and adult [79] Tourette’s syndrome. In addition to pain, excitation of the ACC has also been associated with Tourette’s syndrome [80]. Hence inhibition of ACC AMPAR currents may be a contributing mechanism for the clinical effects of SCH 39166.

Despite the paradoxical pharmacology, there are reasons to believe that inhibition of AMPAR eEPSCs is mediated by D1Rs. First, both SKF 81297 and (±)-SKF 38393 at two different doses have the same effect on eEPSCs and it would be unlikely for two separate agonists at different doses to have the same effect by independent off-target activity. Second, the inhibitory effect of SKF 81297 and (±)-SKF 38393 mimic the effects of dopamine from ACC brain slices in terms of degree of inhibition, synaptic locus of activity and reversibility of the inhibition [30]. Lastly, inhibition of basal transmission by D1R correlates with the observed mechanical sensitivity phenotype in the ACC_D1R-KO mice. These data provide the first evidence that D1Rs in the ACC modulate peripheral sensitivity to mechanical stimuli based on somatic genetic manipulations. In addition, correlational pharmacological data for D1Rs have revealed a potential novel inhibitory mechanism for D1Rs in the cingulate cortex on basal glutamatergic transmission. Further research is imperative to demonstrate the precise mechanism of D1R modulation of mechanical sensitivity, and the functional role of ACC basal transmission in behavior.

## Supplementary information

**Additional file 1 Figure S1**. AMPAR mediated evoked EPSCs in the anterior cingulate cortex. **Figure S2**. AMPAR mediated eEPSC modulation by the DR1 agonist (±)-SKF-38393. **Figure S3**. D1R antagonists do not block D1R agonist effects in ACC slices.

## Data Availability

The datasets used and/or analysed during the current study are available from the corresponding author on reasonable request.

## Abbreviations

ACC: Anterior Cingulate Cortex
ACSF: Artificial cerebrospinal fluid
AMPAR: α-amino-3-hydroxy-5-methyl-4-isoxazolepropionic receptors
CFA: Complete Freund’s adjuvant
D1R: D1 receptor
D2R: D2 receptor
DLET: Dark-light emergence test
EPSCs: Excitatory postsynaptic currents
EPM: Elevated plus maze
GPCR: G protein-coupled receptor subtypes
gRNA: Guide RNA
LTD: Long-term depression
OFT: Open field test
mPFC: Medial prefrontal cortex
SDH: Spinal dorsal horn
sKO: Somatic gene knockout
VTA: Ventral tegmental area
