# Supplementary material for: D1 receptors in the anterior cingulate cortex modulate basal mechanical sensitivity threshold and glutamatergic synaptic transmission
Source: Mol Brain. 2020 Sep 5;13:121. doi: 10.1186/s13041-020-00661-x (PMC7487672; doi:10.1186/s13041-020-00661-x)
Supplement: Supplementary file 1 — Additional file 1 Figure S1. AMPAR mediated evoked EPSCs in the anterior cingulate cortex. Figure S2. AMPAR mediated eEPSC modulation by the DR1 agonist (±)-SKF-38393. Figure S3. D1R antagonists do not block D1R agonist effects in ACC slices. [file 13041_2020_661_MOESM1_ESM.docx]

Supplemental Figures – Dopamine ACC paper


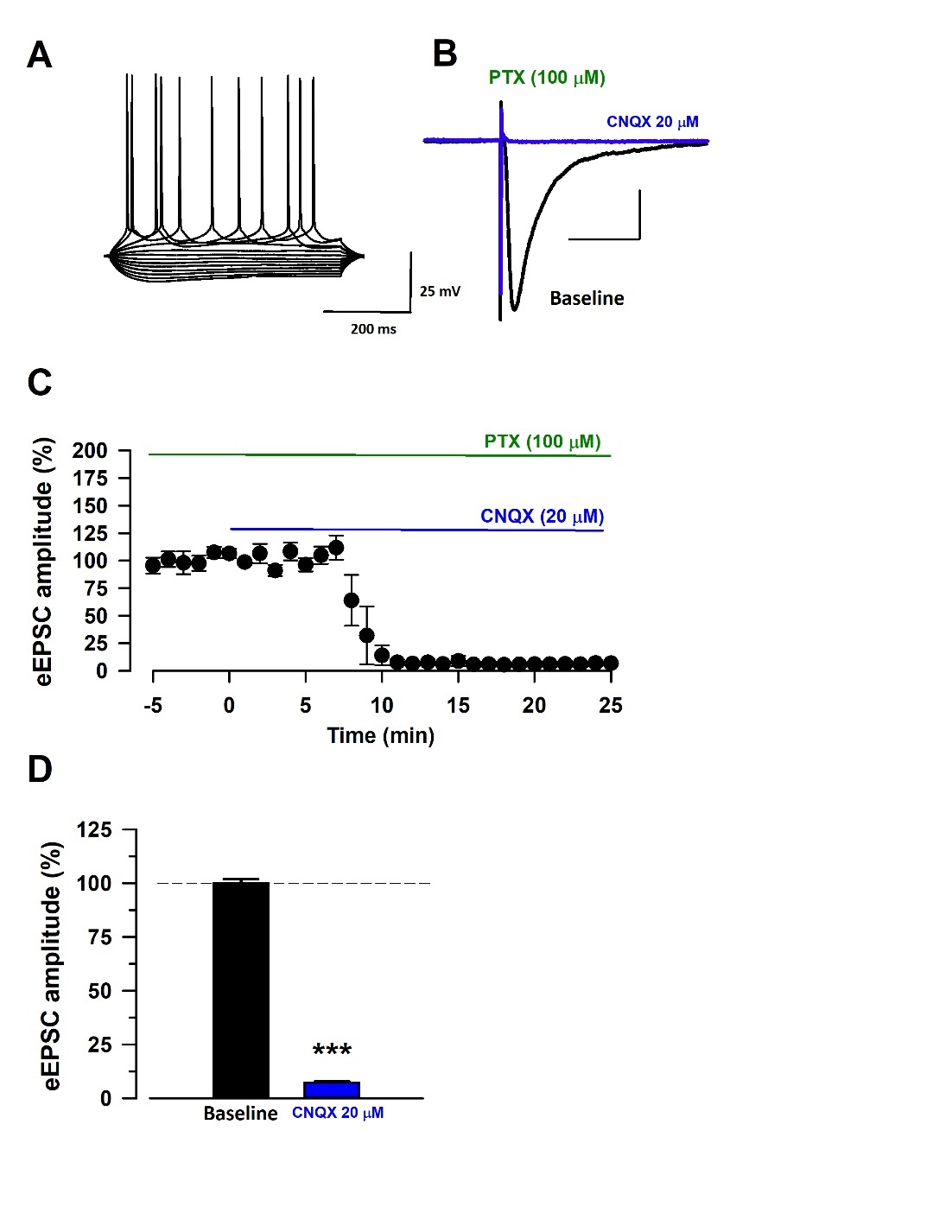


**Supplemental Figure 1. AMPAR mediated evoked EPSCs in the anterior cingulate cortex. (A)** typical action potential firing of principal neurons in the ACC. (**B**) Averaged sample trace of evoked EPSCs in whole-cell patch clamp mode, before and after application of the AMPAR inhibitor CNQX. (**C**) Averaged and normalized data (n=4). (**D**) CNQX 20μM completely blocks eEPSCs in the ACC. (****p*<0.001), paired t-test.


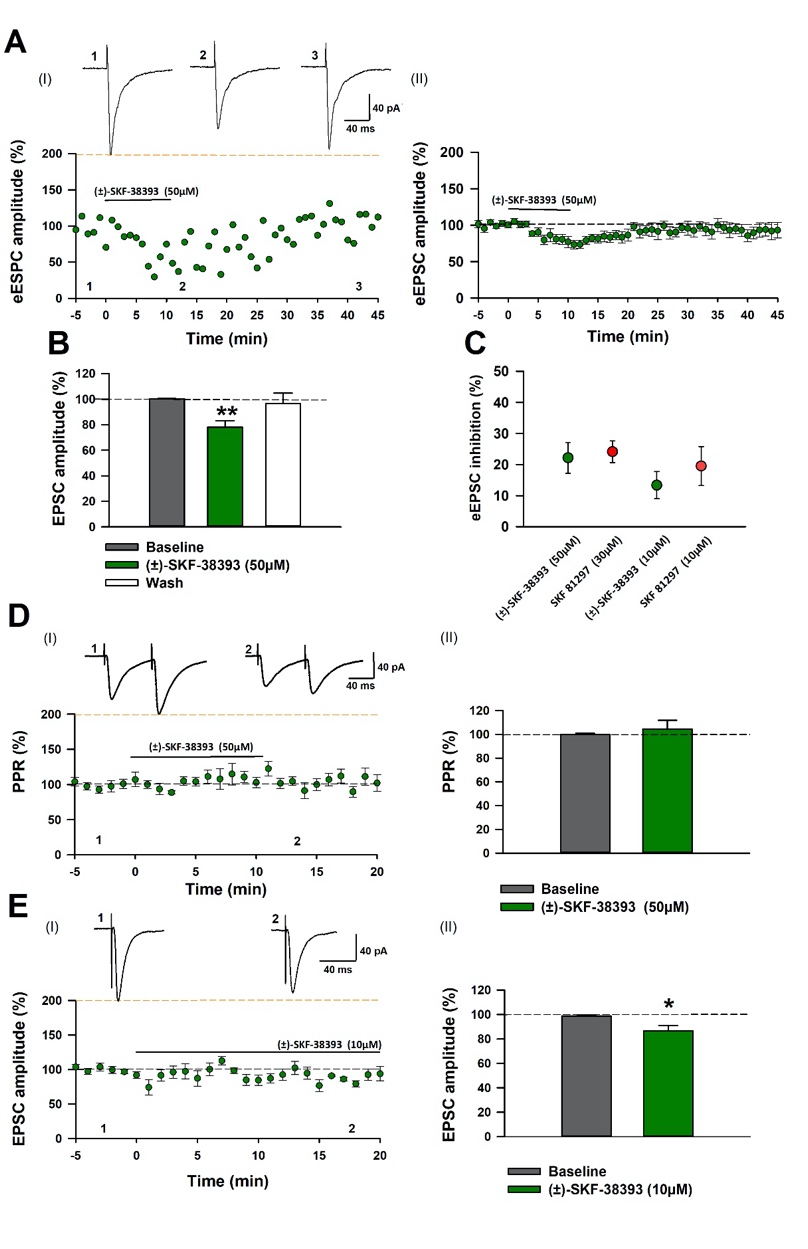


**Supplemental Figure 2. AMPAR mediated eEPSC modulation by the DR1 agonist (±)-SKF-38393. A)** Averaged sample traces of AMPAR eEPSCS and time course of (±)-SKF-38393 (50μM) application. II: averaged and normalized data for time course of (±)-SKF-38393 (50μM) application (n= 10/8 mice). **B)** Transient application of (±)-SKF-38393 (50μM) significantly inhibits eEPSC amplitude with eEPSC values returning to baseline following washout (one-way ANOVA, *F_2,30_*=21.93, *p*<0.001, *n*= 10/8 mice). **C)** No difference in the percent inhibition by SKF 81297 and (±)-SKF-38393 at different concentrations (two-way ANOVA, *main effect of drug:* *F_1,31_*=1.67, *p*=0.21; *main effect of concentration*: *F_1,31_*=3.03, *p*=0.09; *drug x concentration interaction*: *F_1,31_*=0.88, *p*=0.36). **D)** I: Averaged traces for eEPSCs and the averaged and normalized data (n=7/4 mice). II: (±)-SKF-38393 50 μM did not cause a significant change in PPR relative to baseline (paired t-test, *t_6_*=0.57, *p*=0.5883). **(E)** I: Averaged traces of eEPSCs and averaged and normalized values for eEPSC amplitudes (n=5/5 mice). II: Perfusion of (±)-SKF-38393 at 10μM inhibits eEPSC amplitude (paired t-test, *t_4_*=3.39, *p*=0.02).

**
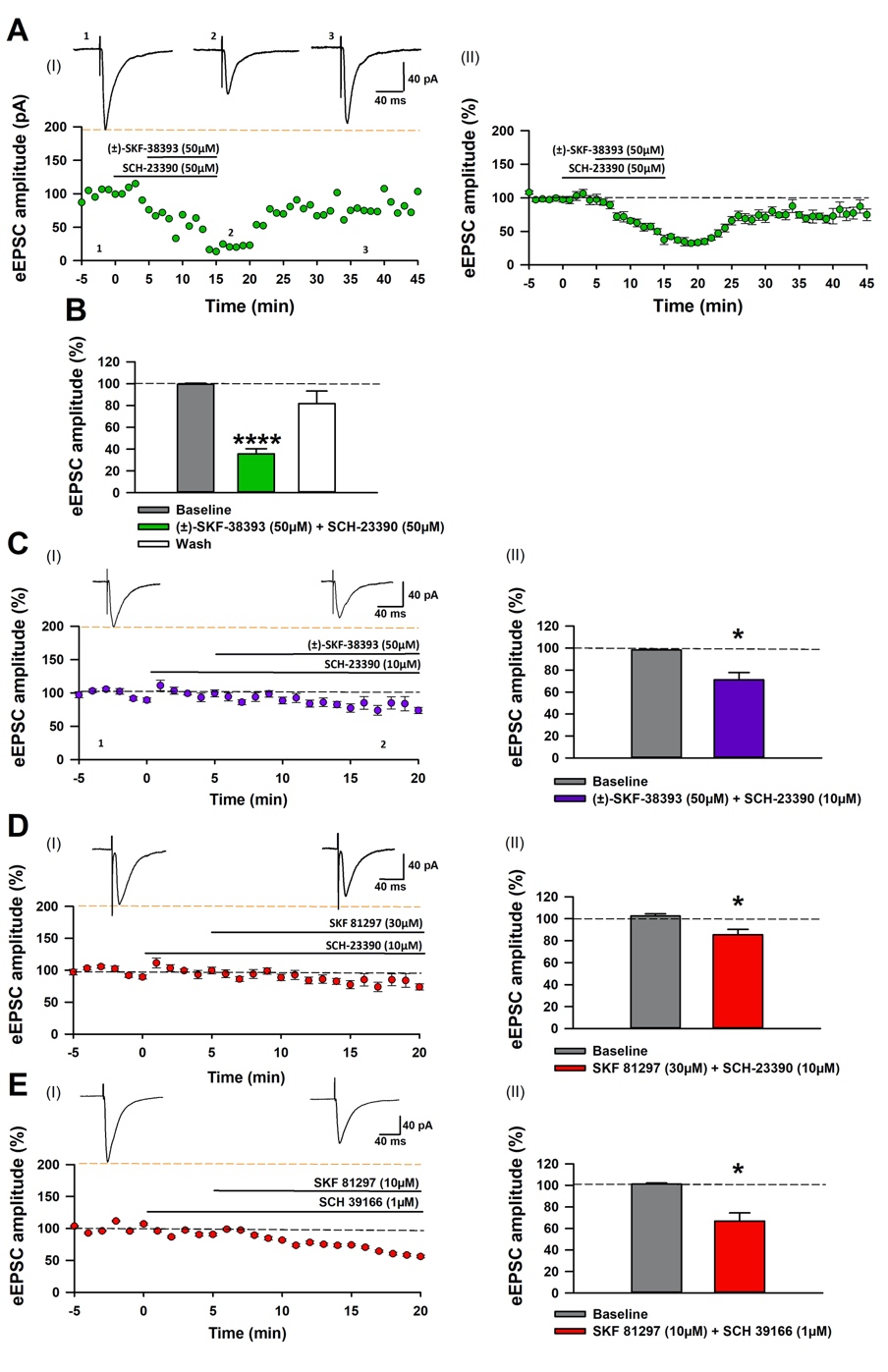
**

**Supplemental Figure 3. Effects of D1R antagonists do not block D1R agonist effects in ACC slices**

**(A)** I: Averaged traces of eEPSC and time course of D1R agonist (±) SKF-38393 (50 µM) and D1R antagonist SCH-23390 (60 µM) administration. II: Averaged and normalized data of eEPSCs (n=6/4 mice). **(B)** Co-application of SCH-23390 (60 µM) and (±)-SKF 38393 (50 µM) significantly inhibits EPSC amplitude, which returned towards baseline following washout (one-way ANOVA, p<0.0001, n =6/4 mice). **(C)** I: Averaged traces of eEPSCs and normalized and averaged data (n=6/5mice). II: Co-application of (±) SKF-38393 50 µM and SCH-23390 10 µM significantly reduced EPSC amplitude (paired t-test, *t_5_*=3.99, *p*=0.01). **(D)** I: Averaged traces of eEPSCs and normalized and averaged data (n=6/5mice)**.** II: Co-application of SCH-23390 (10 µM) with SKF 81297 (30 µM) significantly inhibits eEPSCs (paired t-test, *t_5_*=4.02, *p*=0.01). **(E)** I: Averaged traces of eEPSCs and normalized and averaged data (n=4/3 mice). II: Pre-application of SCH 39166 (1µM) does not block eEPSC inhibition induced by SKF 81297 10 µM (paired t-test, *t_3_*=5.18, *p*=0.01). *p < 0.05, ****p < 0.0001.
